# Feature-token clinical transformer for 24-h landmark mortality risk stratification in critically ill patients with heart failure: development and multicenter external validation

**DOI:** 10.3389/fcvm.2026.1945947

**Published:** 2026-10-15

**Authors:** Mianxian Li, Yuefei Zhou, Chun Li

**Affiliations:** Department of Cardiology, The First Affiliated Hospital of Soochow University, Suzhou, China

**Keywords:** 24-h landmark prediction, critical care, electronic health records, external validation, feature-token transformer, heart failure, mortality prediction

## Abstract

**Background:**

The first 24 h after ICU admission in critically ill patients with heart failure yield heterogeneous EHR evidence across vital signs, laboratory tests, treatments, organ support, and nurse-charted monitoring. Conventional scores and flat-vector ML models may not preserve individual variable identity or context.

**Methods:**

We developed FTCT, encoding each predictor recorded during the first 24 h after ICU admission as a clinically tagged token (value, missingness, identity, group). MIMIC-IV (2008–2019) served for development, validation, and internal testing; eICU-CRD (2014–2015) served for external validation. Included were adults with heart failure, a complete 24-h ICU observation window, and known discharge status. The endpoint was in-hospital all-cause death.

**Results:**

MIMIC-IV included 11,156 stays and 1,484 deaths, partitioned into training (*n* = 7,809), validation (*n* = 1,673), and internal test (*n* = 1,674) sets. eICU-CRD included 9,837 stays and 1,319 deaths. FTCT achieved an AUROC of 0.849 (95% CI, 0.835–0.863) internally and 0.804 (0.788–0.820) externally. Patient-paired AUROC differences vs. XGBoost were 0.027 (0.011–0.043) internally and 0.032 (0.014–0.050) externally. A matched FT-Transformer-style baseline achieved an external AUROC of 0.781 (0.760–0.801). In eICU-CRD, FTCT achieved an AUPRC of 0.296 (0.278–0.314), Brier score of 0.091 (0.085–0.097), and expected calibration error of 0.036 (0.030–0.042). In a *post hoc* cohort-specific descriptive operating analysis targeting approximately 85% specificity, external sensitivity was 0.662 (0.636–0.687), positive predictive value 0.406 (0.385–0.427), negative predictive value 0.942 (0.937–0.947), and F1 score 0.503. Removing feature-token encoding produced the largest ablation loss in external AUROC (−0.031).

**Conclusions:**

FTCT combines established feature-token Transformer modeling with clinical-group embeddings, explicit missingness representation, and group-aware pooling for 24-h landmark mortality risk stratification from structured EHR data recorded during the first 24 h after ICU admission. Its external validation and ablation results support prospective investigation, but do not yet demonstrate clinical utility, workflow integration, admission-time applicability, or autonomous use.

## 1 Introduction

Heart failure is a major cause of hospital admission, critical illness, and death worldwide, with outcomes shaped by heterogeneity in phenotype, organ dysfunction, response to therapy, and comorbidity burden (1–3). In intensive care and coronary care units, the first 24 h after ICU admission are particularly consequential. Hemodynamic instability, oxygen and ventilation requirements, renal dysfunction, diuretic response, exposure to vasoactive drugs, and an evolving fluid-balance trajectory may become apparent before the overall clinical course is established (4–7). This evidence is scattered across the EHR, creating a need for risk-prediction models that can accommodate data recorded during the first 24 h after ICU admission without obscuring the clinical identity of individual variables.

Fixed scores remain important because they rely on variables with which clinicians are already familiar and lead to predictions that are easily communicated. The Acute Decompensated Heart Failure National Registry and GWTG-HF models demonstrated that these routinely collected measures can enable stratification of in-hospital mortality (4, 8). However, external comparisons show that many traditional mortality prediction models in heart failure may offer only moderate discriminatory performance across settings (6). More recent research using MIMIC and eICU data has applied machine-learning methods, such as gradient boosting, to intensive care cohorts of patients with heart failure and has generally demonstrated improved discrimination over fixed scores (9–11). Multimodal models that combine admission notes with structured variables can further enrich representation (12), yet there are substantial differences among institutions in terms of free-text access, documentation practices, and governance. Recent MIMIC-IV-ED evidence also found that GWTG-HF discriminated 30-day mortality better than ADHERE CART, although both scores showed limited performance for ICU-related outcomes, supporting the need for more context-aware assessment when physiology and treatment response evolve (13).

Consequently, structured EHR modeling remains clinically relevant, but it poses challenges that are not well addressed when a patient is represented as a single flat vector. Records from the first 24 h after ICU admission contain continuous measurements, binary intervention flags, categorical diagnosis codes, severity scores, monitoring-derived variables, and informative missingness. Measurement itself can imply clinician concern, and the clinical meaning of a variable depends on its relationship to other variables in the record; for example, elevated creatinine accompanied by low urine output, vasopressor exposure, or mechanical ventilation has a different meaning than when it appears in isolation. The literature on deep EHR modeling has emphasized both the potential and the complexity of learning from heterogeneous and incomplete clinical data (14–18). Although deidentified critical-care databases and benchmark studies have enabled reproducible development (19–21), transport across institutions remains difficult. In practice, EHRs differ across institutions because of differences in patient populations, measurement practices, coding practices, and clinical workflows. At the representational level, attention-based tabular models offer an alternative to direct aggregation. The feature-token approach keeps input variables as learnable units and uses self-attention to model interactions, as previously described in FT-Transformer and related tabular architectures (22, 23); feature tokenization itself is therefore not claimed as a novel contribution. However, generic tabular representations do not automatically encode clinical grouping, preserve missingness context, or establish validity in an independent multicenter cohort. Architectural traceability should also not be conflated with validated clinical interpretability or clinical workflow benefit. Accordingly, we developed the Feature-Token Clinical Transformer (FTCT) for 24-h landmark prediction of in-hospital mortality among critically ill adults with heart failure. FTCT converts each retained EHR field recorded during the first 24 h after ICU admission into a token carrying value, missingness, variable-identity, and clinical-group information and uses a learned risk token to capture cross-feature interactions for patient-level prediction. Our work presents four primary contributions. First, we defined a harmonized first-24-h cohort for in-hospital mortality prediction, with single-center MIMIC-IV development and multicenter eICU-CRD validation. Second, we evaluated the combination of clinical-group embeddings, explicit missingness representation, group-aware pooling, and cross-database harmonization within a feature-token Transformer. Third, we examined discrimination, precision–recall performance, probability error, calibration, and the *post hoc* high-specificity operating profiles of our approach against multiple machine-learning and clinical-score comparators. Finally, we tested the proposed representation through prespecified ablation, missingness, observation-window, cross-database common-feature, and hospital-reweighting analyses.

## 2 Materials and methods

### 2.1 Study design and data sources

We conducted a retrospective study of 24-h landmark mortality risk stratification in adult ICU/CCU patients with heart failure, using MIMIC-IV for single-center model development and internal testing and eICU-CRD for independent multicenter external validation. MIMIC-IV contains demographic information, diagnoses, laboratory test results, medications, bedside measurements, procedures, and outcomes for patients hospitalized in the critical-care units of Beth Israel Deaconess Medical Center in Boston. eICU-CRD is a multicenter ICU database containing corresponding information, including vital signs, laboratory tests, medications, diagnoses, Acute Physiology and Chronic Health Evaluation (APACHE) components, treatment records, care-plan information, nurse-charted variables, intake and output records, and patient outcomes. MIMIC-IV version 2.2 covers admissions recorded during 2008–2019, whereas eICU-CRD version 2.0 contains encounters from 2014 to 2015. The calendar periods partially overlap; therefore, eICU-CRD provides cross-institutional rather than forward temporal validation. MIMIC-IV is derived from Beth Israel Deaconess Medical Center, whose records were not contributed to eICU-CRD; the databases share no source institution or analytic patient record, and no cross-database patient linkage was performed.

### 2.2 Reporting framework and methodological appraisal

This study was reported in accordance with TRIPOD + AI, and the model-development and external validation procedures were appraised using PROBAST + AI (24, 25).

### 2.3 Study population and endpoint

Eligible patients were aged 18 years or older. In MIMIC-IV, heart failure was identified from any diagnosis sequence using ICD-9-CM category 428 or ICD-10-CM category I50, including all respective subcodes. In eICU-CRD, heart failure was identified using ICD-9-CM category 428 or explicit active-diagnosis labels for heart failure, congestive heart failure, or CHF, including Primary, Major, and Other priorities. Eligibility was not restricted to the principal diagnosis. The complete database-specific definitions and code ranges are provided in Supplementary Table S1. For patients with multiple ICU admissions, only the first ICU admission was retained. Stays shorter than 24 h and stays without known hospital discharge status were excluded. Diagnosis information was used only for retrospective cohort ascertainment, was not assumed to be available at the 24-h landmark, and was not included as a predictor.

The primary endpoint was all-cause death before hospital discharge, while survival to hospital discharge was the negative outcome. All predictors were restricted to information available within the first 24 h after ICU admission. Discharge variables, outcome-derived fields, and interventions or measurements occurring after the observation window were excluded. The cohort definition, endpoint, MIMIC-IV training, validation, and internal test split, the role of eICU-CRD as the external validation cohort, and the confidence-interval procedures were prespecified before final evaluation of the held-out cohorts.

Applying the inclusion criteria yielded 11,156 eligible MIMIC-IV stays, including 1,484 deaths and 9,672 survivors. Patient-level stratified partitioning produced training, validation, and internal test sets containing 7,809, 1,673, and 1,674 stays, respectively. eICU-CRD included 9,837 eligible stays, comprising 1,319 deaths and 8,518 survivors. No eICU-CRD record was used for model fitting, hyperparameter tuning, feature selection, preprocessing estimation, model-development threshold selection, or probability calibration.

### 2.4 Feature extraction and preprocessing

Structured variables were extracted from the first 24 h after ICU admission and organized into eight clinical groups: demographics (age, sex, weight, and height); severity measures (SOFA and Acute Physiology Score III); comorbidities (chronic kidney disease, diabetes mellitus, hypertension, atrial fibrillation, severe liver disease, chronic obstructive pulmonary disease, and myocardial infarction); vital signs (heart rate, respiratory rate, SpO2, systolic blood pressure, and mean arterial pressure); laboratory tests (blood counts, albumin, liver enzymes, bilirubin, blood urea nitrogen, creatinine, electrolytes, anion gap, bicarbonate, international normalized ratio, activated partial thromboplastin time, and prothrombin time); treatments (vasopressors, inotropes, loop diuretics, beta blockers, angiotensin-converting enzyme inhibitors, and angiotensin receptor blockers); organ support (mechanical ventilation and renal replacement therapy); and nursing monitoring (urine output and harmonized intake/output summaries). Preprocessing was fitted exclusively on the MIMIC-IV training set and subsequently frozen. Continuous features were winsorized and standardized using limits, means, and standard deviations derived solely from the training set. Categorical vocabularies were also derived from the training set, and categories not observed during training were mapped to an unknown category. Missing values were imputed using a random-forest imputer fitted exclusively on the training set (16). A binary missingness indicator was added to every retained feature so that missingness remained explicit after imputation. For variables collected at admission, the first available value within the observation window was used; for repeatedly measured variables, clinically standard first-24-h summaries were calculated. Cross-database definitions, units, and categories were harmonized. The same feature dictionary and, where applicable, the same preprocessing objects were used for FTCT and each machine-learning comparator.

### 2.5 FTCT architecture

FTCT is a patient-level Transformer designed for heterogeneous structured EHR data. Continuous variables were projected linearly into token space. Binary treatments, comorbidities, and categorical variables used embedding tables. The variable-identity embedding distinguishes fields within a group, while the clinical-group embedding identifies demographics, severity scores, comorbidities, vital signs, laboratory tests, treatments, organ support, or nursing monitoring.

A learned risk token was prepended to the feature-token sequence. After the final block, the updated risk token was concatenated with an attention-weighted mean of clinical-group summaries. The resulting representation was projected to 128- and 64-dimensional hidden layers, with GELU activation, dropout, layer normalization, and a final sigmoid unit producing the probability of in-hospital death. The architecture was fixed before internal test and external validation evaluation.

### 2.6 Model training and selection

FTCT was optimized with weighted binary cross-entropy: L=−[wylog(p)+(1−y)log(1−p)], where *y* is the death indicator, *p* is the predicted probability, and *w* is the positive-class weight. AdamW optimization used a learning rate of 3 × 10^−4^, weight decay of 1 × 10^−4^, cosine learning-rate decay, and five warmup epochs. Batch size was 256 and dropout was 0.16. Training continued for up to 80 epochs with early stopping after 12 epochs without validation-AUROC improvement. Mixed-precision training was performed on a single A100-class GPU. MIMIC-IV validation data selected hyperparameters; frozen weights were then used for internal testing and eICU-CRD external validation.

### 2.7 Comparison models

All five comparators used the same prediction window, endpoint, patient partitions, and harmonized predictor set. XGBoost represents gradient-boosted decision trees (26); LightGBM represents histogram-based leaf-wise boosting; random forest represents bagged tree ensembles (27); and elastic-net logistic regression represents a regularized linear prediction model (28). The boosting models were tuned for depth, learning rate, subsampling, and the number of boosting iterations based on MIMIC-IV validation performance. The random forest used 500 trees, with feature-subsampling and node-size settings tuned using the validation data. The elastic-net mixing and penalty parameters were selected based on validation AUROC.

A matched FT-Transformer-style controlled baseline was implemented using the same patients, predictors, partitions, preprocessing, Transformer depth, training budget, and model-selection procedure. It retained generic feature tokenization and self-attention but omitted FTCT-specific clinical-group embeddings, dedicated missingness embeddings, and group-aware pooling (23).

GWTG-HF was chosen as the clinical reference for this study because it is a validated mortality score for hospitalized patients with heart failure and recent MIMIC-IV-ED evidence found better short-term mortality discrimination than ADHERE CART (8, 13). Its seven components were harmonized across databases using prespecified measurement timing, units, COPD definitions, race/ethnicity mapping, and missing-data rules (Supplementary Table S4). Sporadically missing components were handled using the imputation procedure fitted exclusively in the MIMIC-IV training set. Because the Brier score and ECE require probability estimates, a univariable logistic calibration model mapped the total score to in-hospital mortality probability in the MIMIC-IV training set. The imputation and score-to-probability mapping were then applied unchanged to all held-out cohorts.

### 2.8 Evaluation and statistical analysis

We used AUROC as the primary discrimination metric and reported AUPRC because the event prevalence was approximately 13% (29). We evaluated probability performance using the Brier score and ECE; ECE was calculated separately for each model and cohort using 10 equal-frequency bins (30, 31). For *post hoc* descriptive threshold-based evaluation, each held-out cohort used its own outcome-informed threshold corresponding to approximately 85% specificity (OP85SPEC). This yielded sensitivity, specificity, PPV, NPV, F1 score, accuracy, and auditable class counts. Because the threshold was selected separately using outcome labels from the corresponding held-out cohort, these values represent *post hoc* cohort-specific operating profiles and do not constitute fixed-threshold external validation performance or validation of a shared deployment threshold. Patient-level nonparametric bootstrap resampling with 1,000 prespecified replicates was used to generate percentile 95% CIs for AUROC, AUPRC, Brier score, ECE, calibration-in-the-large, and calibration slope. A replicate was unsuccessful if only one outcome class was present, a metric could not be calculated, or a non-finite estimate was produced; all replicates were successful. FTCT and XGBoost were compared using the same patient indices in 1,000 paired bootstrap replicates; detailed difference definitions and results are provided in Supplementary Methods S1 and Table S2. Wilson binomial intervals were calculated for sensitivity, specificity, positive predictive value, negative predictive value, and accuracy using the corresponding confusion-matrix denominators; the F1 score was reported as a point estimate. Continuous baseline variables were summarized as mean [standard deviation (SD)] or median [interquartile range (IQR)], as appropriate, and categorical variables as *n* (%). The unit of analysis was the ICU stay. Calibration plots used 10 equal-frequency risk groups with Wilson 95% CIs for observed mortality, and predicted-risk distributions were summarized in both cohorts. No probability recalibration was performed in eICU-CRD. Net reclassification improvement and decision-curve analysis were not performed because no validated risk categories, prespecified deployment threshold range, or model-triggered clinical intervention was defined. We evaluated eICU-CRD only after model development and selection were complete. Evaluation focused on external validation, calibration, and transparent reporting of model operating characteristics, which are central to clinical prediction-model assessment (24, 25).

### 2.9 Ablation, robustness, sensitivity, and efficiency analyses

We conducted prespecified ablation studies in which one of the following FTCT components was removed or modified at a time: feature-token encoding, clinical-group embeddings, attention depth, missingness embedding, the nursing-monitoring group, or learned risk-token pooling. Each ablation model predicted the same endpoint in the same external validation cohort.

Frozen-model robustness and sensitivity analyses included restricting inputs to the MIMIC-IV/eICU-CRD common-feature intersection, applying a 20% stricter variable-missingness criterion, truncating the observation window to 12 h or 6 h, masking 15% of observations, and performing equal-hospital-weighted hospital-cluster bootstrap resampling. Masked observations followed the same imputation policy as in the main analyses and retained their missingness channels. The final eICU-CRD cohort comprised 9,837 patients from 208 hospitals. In each of 1,000 hospital-cluster bootstrap replicates, 208 hospitals were sampled with replacement, all eligible patients within each selected hospital were retained together, and each hospital contributed equal total weight; all replicates were successful (Supplementary Methods S2). Efficiency was summarized by the number of parameters, multiply-accumulate operations per patient, and inference latency. Latency was measured on one NVIDIA A100 40-GB GPU using Python 3.10, PyTorch 2.1.2 + cu121, CUDA 12.1, and FP16 automatic mixed precision. After 50 untimed warm-up iterations, 200 forward passes were measured with synchronized CUDA events for batch sizes of 1 and 1,000. Inputs were preprocessed and resident in GPU memory; database queries, feature extraction, preprocessing, disk input/output, host-to-device transfer, and CPU time were excluded (Supplementary Methods S3). No test-time augmentation was used, and no model was retrained using eICU-CRD.

## 3 Results

### 3.1 Cohort construction and first-24-h clinical profile

The study included adult ICU/CCU admissions with heart failure, a complete first-24-h observation window, and known hospital discharge status. MIMIC-IV contained 11,156 eligible first ICU admissions, including 1,484 in-hospital deaths (13.3%). Patient-level partitioning produced a training cohort of 7,809 ICU admissions with 1,039 deaths, a validation cohort of 1,673 admissions with 222 deaths, and an internal test cohort of 1,674 admissions with 223 deaths. The independent eICU-CRD external validation cohort included 9,837 ICU admissions, of which 1,319 (13.4%) resulted in in-hospital death. No eICU record was used for model fitting, feature selection, imputation, scaling, calibration fitting, or hyperparameter selection. MIMIC-IV covered 2008–2019 and eICU-CRD covered 2014–2015. No source institution or analytic patient record was shared between the databases, and no cross-database linkage was performed.

Figure 1 presents the clinical rationale and workflow of the study. In panel A, FTCT is conceptualized as a retrospective 24-h landmark mortality-risk estimation model that organizes structured evidence from EHR data recorded during the first 24 h after ICU admission rather than as an admission-time alert. Panel B illustrates the separation of model development in MIMIC-IV from multicenter external validation in eICU-CRD, with the model weights and preprocessing objects derived from MIMIC-IV frozen before their application to eICU-CRD. Panel C outlines the standardized pipeline from eligibility assessment and eight clinical evidence groups within the first-24-h window, through training-set-fitted preprocessing and feature-token construction, to in-hospital mortality probability estimation. This diagram highlights the prediction time point and the prohibition against using information generated after the first 24-h window or at hospital discharge.

**Figure 1:**
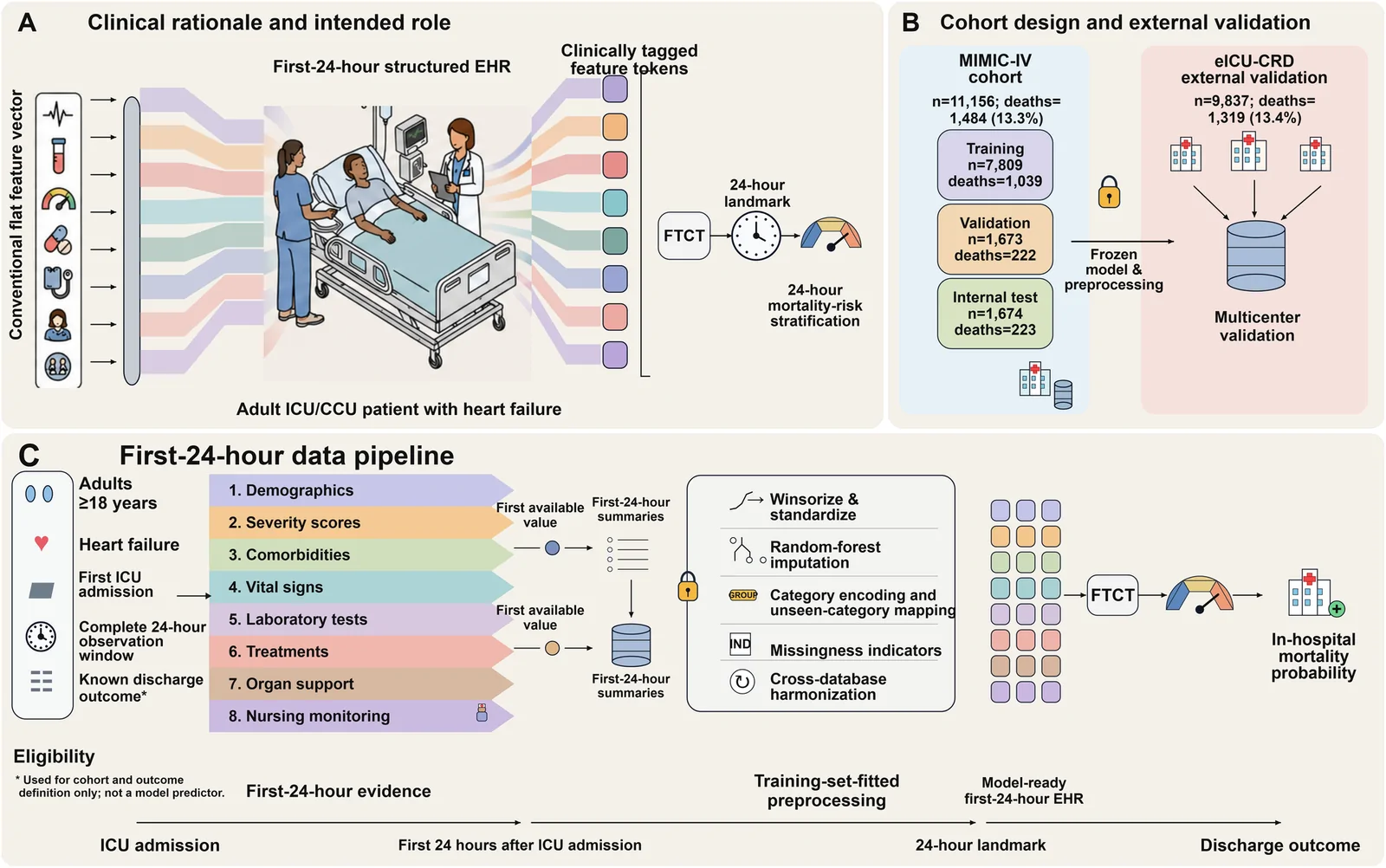
Clinical rationalen, cohort design, and first-24-h data pipeline. **(A)** FTCT converts structured EHR evidence recorded during the first 24 h after ICU admission into clinically tagged feature tokens and generates an in-hospital mortality risk estimate at the 24-h landmark. **(B)** MIMIC-IV was used for development, validation, and internal testing; the frozen model and preprocessing were applied without refitting or recalibration to eICU-CRD for multicenter external validation. **(C)** Eligibility, eight clinical evidence groups, training-set-fitted preprocessing, feature-token construction, and mortality-risk readout are shown. Heart-failure diagnosis and discharge outcome were used only for cohort and outcome definition, not as model predictors.

Baseline characteristics were similar across the three MIMIC-IV partitions and broadly similar to those in eICU-CRD (Table 1). Mean age ranged from 71.6 to 72.4 years, the proportion of male patients ranged from 53.1% to 54.1%, and the mean first-24-h SOFA score ranged from 5.5 to 5.8. In the external cohort, the frequencies of chronic kidney disease, diabetes, atrial fibrillation, mechanical ventilation, vasopressor use, and loop-diuretic use were slightly lower, but event prevalence was nearly identical. Median first-24-h urine output was 1,160 mL in eICU-CRD and ranged from 1,185 to 1,220 mL across the MIMIC-IV partitions; median creatinine and blood urea nitrogen were also similar. These findings indicate that external validation was conducted in a clinically similar but institutionally distinct population rather than in a resampled subset of the development database.

**Table 1:** Cohort characteristics and first-24-h clinical information.

Characteristic	MIMIC-IV training (*n* = 7,809)	MIMIC-IV validation (*n* = 1,673)	MIMIC-IV internal test (*n* = 1,674)	eICU-CRD external validation (*n* = 9,837)
Death before hospital discharge, *n* (%)	1,039 (13.3)	222 (13.3)	223 (13.3)	1,319 (13.4)
Age, years, mean (SD)	72.1 (12.9)	72.4 (12.7)	72.2 (13.1)	71.6 (12.5)
Male sex, *n* (%)	4,221 (54.1)	894 (53.4)	901 (53.8)	5,224 (53.1)
SOFA score during the first 24 h after ICU admission, mean (SD)	5.7 (3.1)	5.8 (3.0)	5.7 (3.2)	5.5 (3.0)
GWTG-HF score, median (IQR)	39 (32–48)	40 (32–49)	39 (31–48)	38 (31–47)
Chronic kidney disease, *n* (%)	2,574 (33.0)	552 (33.0)	549 (32.8)	3,108 (31.6)
Diabetes mellitus, *n* (%)	3,052 (39.1)	657 (39.3)	646 (38.6)	3,732 (37.9)
Atrial fibrillation, *n*n(%)	2,948 (37.8)	621 (37.1)	635 (37.9)	3,548 (36.1)
Mechanical ventilation during the first 24 h after ICU admission, *n* (%)	2,217 (28.4)	469 (28.0)	474 (28.3)	2,705 (27.5)
Vasopressor exposure during the first 24 h after ICU admission, *n* (%)	1,983 (25.4)	418 (25.0)	431 (25.7)	2,390 (24.3)
Loop diuretic exposure during the first 24 h after ICU admission, *n* (%)	3,431 (43.9)	724 (43.3)	731 (43.7)	4,121 (41.9)
Urine output during the first 24 h after ICU admission, mL, median (IQR)	1,220 (720–1,910)	1,185 (700–1,870)	1,205 (705–1,895)	1,160 (690–1,830)
Serum creatinine, mg/dL, median (IQR)	1.39 (0.98–2.10)	1.42 (1.00–2.12)	1.40 (0.99–2.11)	1.36 (0.95–2.05)
Blood urea nitrogen, mg/dL, median (IQR)	31 (22–47)	32 (22–48)	31 (21–47)	30 (21–45)
Missingness indicators retained, *n* (%)	7,809 (100)	1,673 (100)	1,674 (100)	9,837 (100)

Data are presented as mean (SD), median (IQR), or *n* (%). GWTG-HF, Get With The Guidelines-Heart Failure; IQR, interquartile range; SOFA, Sequential Organ Failure Assessment.

Figure 2 shows sequential participant selection and all exclusion counts for MIMIC-IV and eICU-CRD.

**Figure 2:**
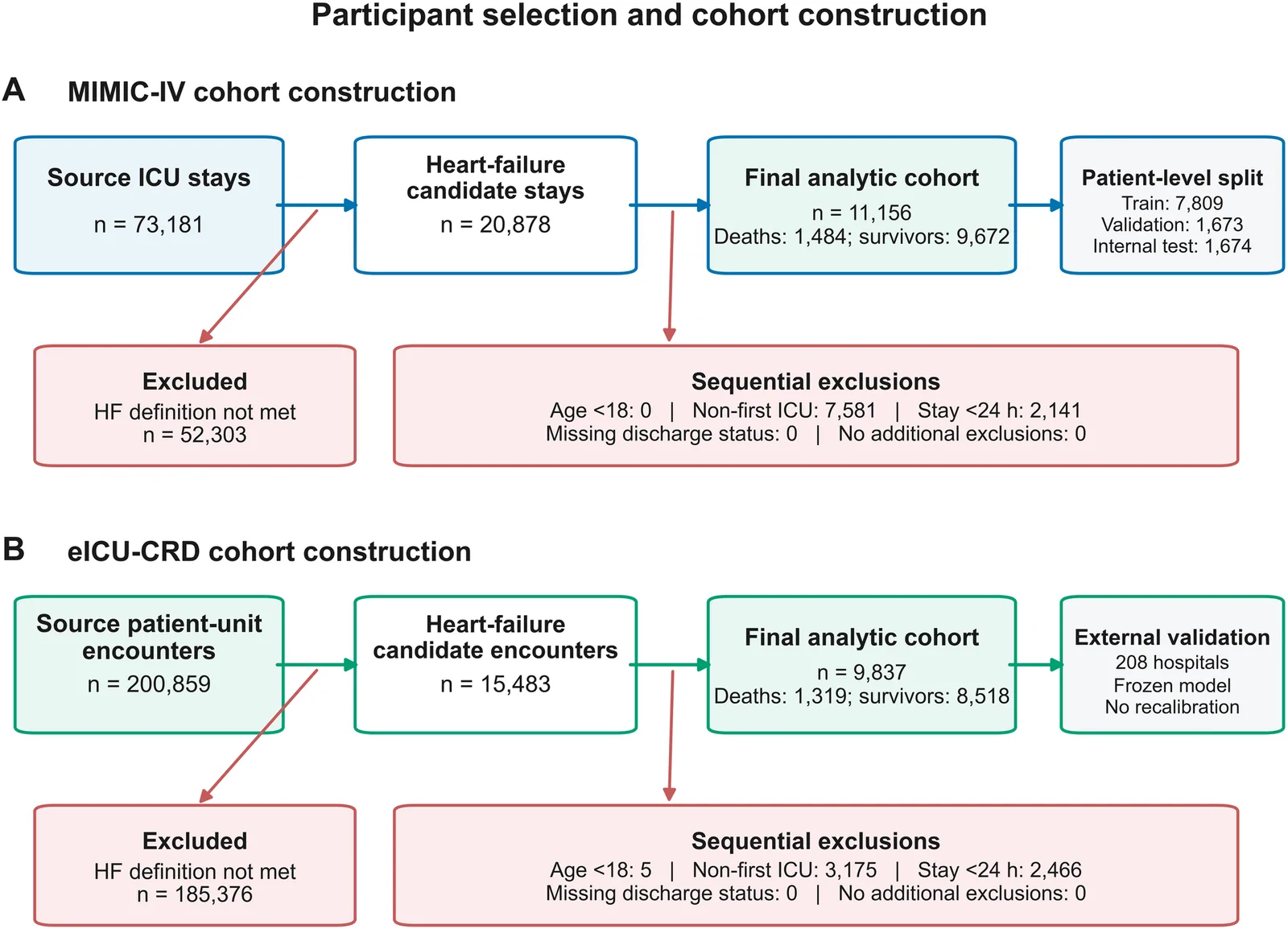
Participant selection and cohort construction. Participant selection is shown for MIMIC-IV **(A)** and eICU-CRD **(B)** Exclusions were applied sequentially for the database-specific heart-failure definition, age younger than 18 years, non-first ICU admission, ICU stay shorter than 24 h, missing hospital discharge status, and additional prespecified criteria. The final MIMIC-IV cohort was partitioned at the patient level into training, validation, and internal test sets; eICU-CRD was reserved for external validation.

### 3.2 Feature-token representation and risk architecture

Figure 3 outlines the complete FTCT architecture evaluated in the held-out cohorts. In panel A, each variable recorded during the first 24 h after ICU admission appears as a unique token annotated by its clinical evidence group. This is achieved by combining the observed or imputed variable value with explicit missingness, variable-identity, and clinical-group embeddings, meaning that a missing laboratory measurement is represented differently from one that was measured and found to be normal. These groups include demographics, severity measures, comorbidities, vital signs, laboratory tests, treatment exposures, organ support, and nursing-monitoring data.

**Figure 3:**
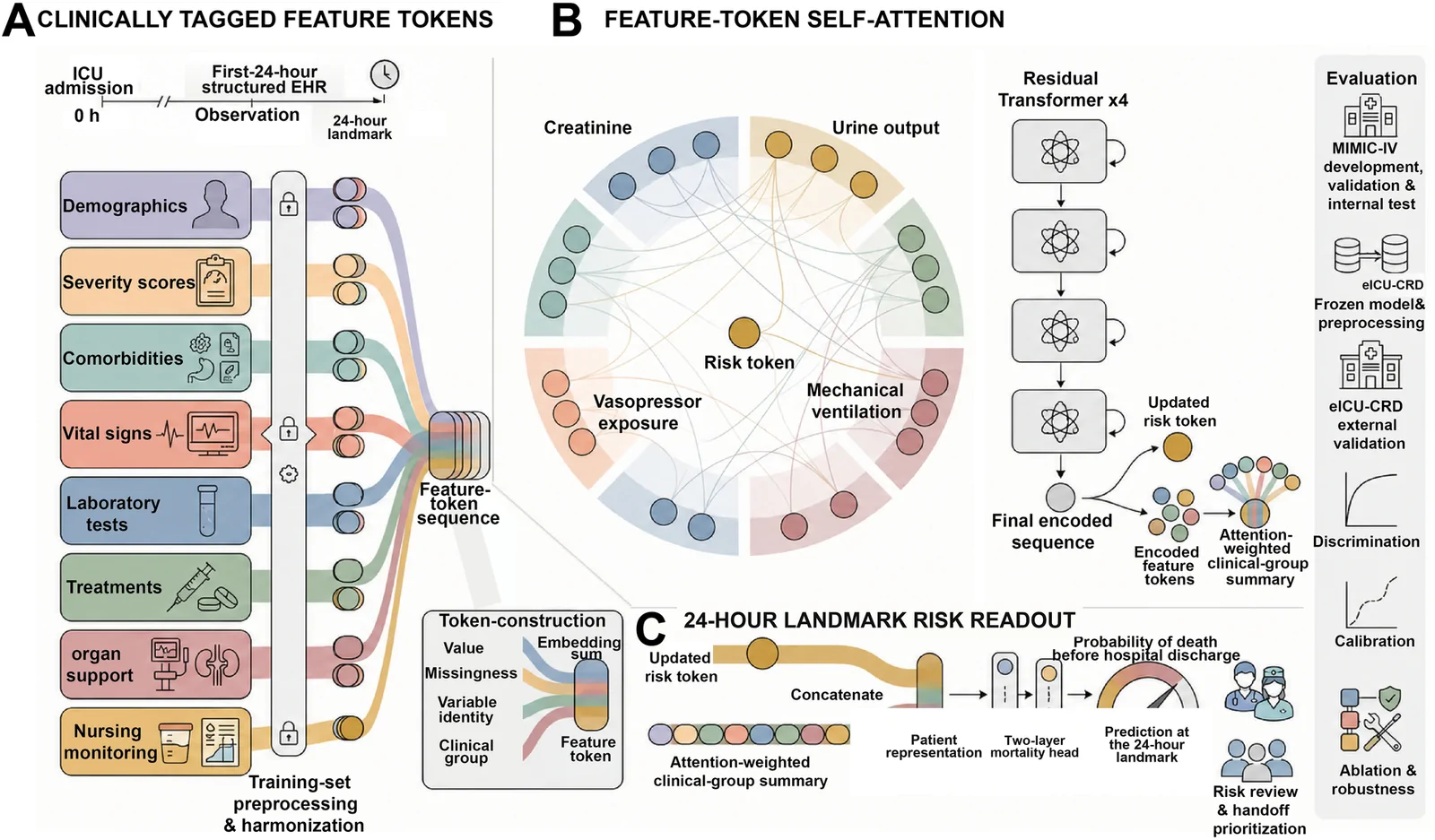
FTCT architecture for 24-h landmark in-hospital mortality prediction. **(A)** Clinically tagged feature-token construction from structured EHR data recorded during the first 24 h after ICU admission. **(B)** Feature-token self-attention with four residual Transformer blocks and a learned risk token. **(C)** Integration of the updated risk token and attention-weighted clinical-group summary. The same frozen architecture was evaluated in the MIMIC-IV internal test and eICU-CRD external validation cohorts.

Panel B shows four residual Transformer blocks in which feature tokens exchange information through multi-head self-attention and update a learned risk token. This allows the model to capture complex interactions among multiple signals, including, for example, the combination of renal dysfunction, low urine output, vasoactive-drug exposure, and mechanical ventilation, rather than considering each signal independently. Panel C shows the 24-h landmark readout, which combines the final risk token with an attention-weighted clinical-group summary and passes the resulting representation through a two-layer prediction head to estimate the probability of in-hospital death. The internal test and external validation pathways remained separate from model development. The prespecified model weights and preprocessing objects were frozen before application to the MIMIC-IV internal test and eICU-CRD external validation cohorts, with no further model training.

### 3.3 Internal and external model performance

In both held-out cohorts, FTCT had the most favorable point estimates among the evaluated models (Table 2). In the MIMIC-IV internal test cohort, FTCT achieved an AUROC of 0.849 (95% CI: 0.835–0.863), compared with 0.822 (0.805–0.839) for XGBoost. The patient-paired AUROC difference was 0.027 (95% CI: 0.011–0.043). The internal AUPRC of FTCT was 0.324 (0.308–0.340), and its Brier score and ECE were 0.085 (0.079–0.091) and 0.033 (0.027–0.039), respectively. Detailed paired differences are reported in Supplementary Table S2; comparisons with the remaining models were interpreted descriptively.

**Table 2:** Discrimination and probability-performance metrics.

Dataset	Method	AUROC (95% CI)	AUPRC (95% CI)	Brier score (95% CI)	ECE (95% CI)
MIMIC-IV internal test	FTCT	0.849 (0.835–0.863)	0.324 (0.308–0.340)	0.085 (0.079–0.091)	0.033 (0.027–0.039)
MIMIC-IV internal test	XGBoost	0.822 (0.805–0.839)	0.257 (0.238–0.276)	0.099 (0.092–0.106)	0.046 (0.039–0.053)
MIMIC-IV internal test	LightGBM	0.816 (0.796–0.836)	0.251 (0.229–0.273)	0.101 (0.093–0.109)	0.048 (0.040–0.056)
MIMIC-IV internal test	Random forest	0.805 (0.799–0.811)	0.242 (0.234–0.250)	0.102 (0.098–0.106)	0.054 (0.050–0.058)
MIMIC-IV internal test	Elastic-net logistic regression	0.779 (0.772–0.786)	0.222 (0.213–0.231)	0.105 (0.101–0.109)	0.056 (0.052–0.060)
MIMIC-IV internal test	GWTG-HF score	0.690 (0.676–0.704)	0.176 (0.160–0.192)	0.118 (0.112–0.124)	0.070 (0.064–0.076)
eICU-CRD external validation	FTCT	0.804 (0.788–0.820)	0.296 (0.278–0.314)	0.091 (0.085–0.097)	0.036 (0.030–0.042)
eICU-CRD external validation	XGBoost	0.772 (0.753–0.791)	0.258 (0.237–0.279)	0.100 (0.093–0.107)	0.051 (0.044–0.058)
eICU-CRD external validation	LightGBM	0.766 (0.744–0.788)	0.252 (0.228–0.276)	0.101 (0.093–0.109)	0.054 (0.046–0.062)
eICU-CRD external validation	Random forest	0.759 (0.751–0.767)	0.244 (0.234–0.254)	0.105 (0.101–0.109)	0.060 (0.056–0.064)
eICU-CRD external validation	Elastic-net logistic regression	0.725 (0.716–0.734)	0.219 (0.208–0.230)	0.108 (0.104–0.112)	0.063 (0.059–0.067)
eICU-CRD external validation	GWTG-HF score	0.649 (0.633–0.665)	0.169 (0.151–0.187)	0.123 (0.117–0.129)	0.077 (0.071–0.083)

In the eICU-CRD external validation cohort, FTCT achieved an AUROC of 0.804 (95% CI, 0.788–0.820), compared with 0.772 (0.753–0.791) for XGBoost. The patient-paired AUROC difference was 0.032 (95% CI, 0.014–0.050). FTCT achieved an AUPRC of 0.296 (0.278–0.314), Brier score of 0.091 (0.085–0.097), and ECE of 0.036 (0.030–0.042). Detailed paired differences are reported in Supplementary Table S2. FTCT had more favorable point estimates than the other evaluated comparators, but those comparisons were descriptive.

Figure 4 presents calibration and predicted-risk distributions in both held-out cohorts. Calibration-in-the-large was −0.164 (95% CI: −0.355 to 0.023) internally and 0.297 (0.215–0.376) externally; corresponding calibration slopes were 0.767 (0.673–0.885) and 0.609 (0.567–0.649). External calibration error was risk-dependent: mortality was modestly underpredicted in several low-to-intermediate-risk groups and overpredicted in the highest-risk group, while the cohort-level mean showed modest underprediction (mean predicted risk 0.113 vs. observed risk 0.134). Predicted-risk summaries are provided in Supplementary Table S3. Figure 5 shows the model-specific performance estimates and confidence intervals.

**Figure 4:**
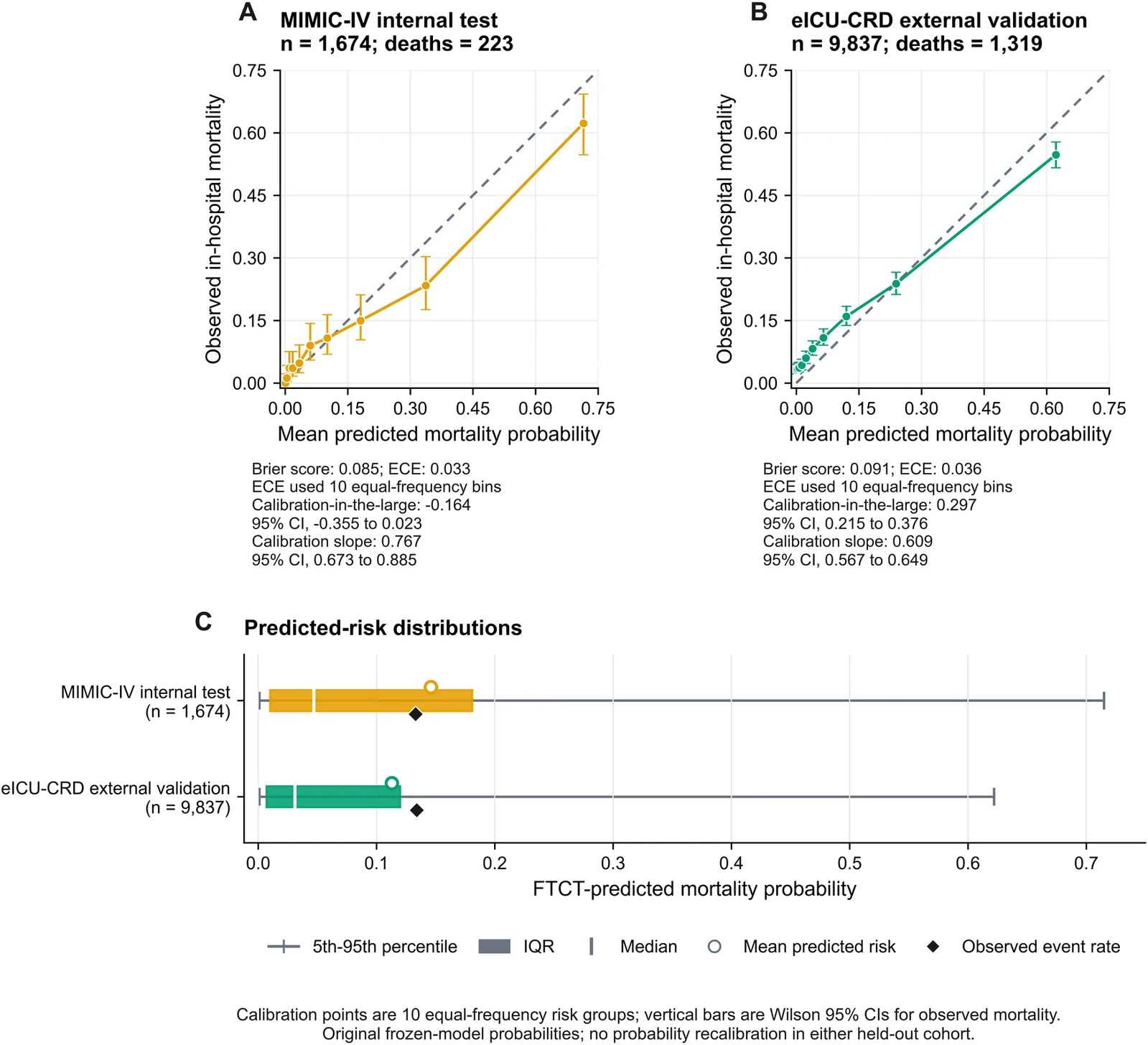
Calibration and predicted-risk distributions in held-out cohorts. **(A)** MIMIC-IV internal test cohort (*n* = 1,674; 223 in-hospital deaths). **(B)** eICU-CRD external validation cohort (*n* = 9,837; 1,319 in-hospital deaths). Points represent 10 equal-frequency risk groups; vertical error bars are Wilson 95% confidence intervals for observed in-hospital mortality. Calibration-in-the-large and calibration slope are reported with patient-level percentile-bootstrap 95% confidence intervals. **(C)** Predicted-risk distributions in both cohorts. The frozen FTCT probabilities were not recalibrated in eICU-CRD.

**Figure 5:**
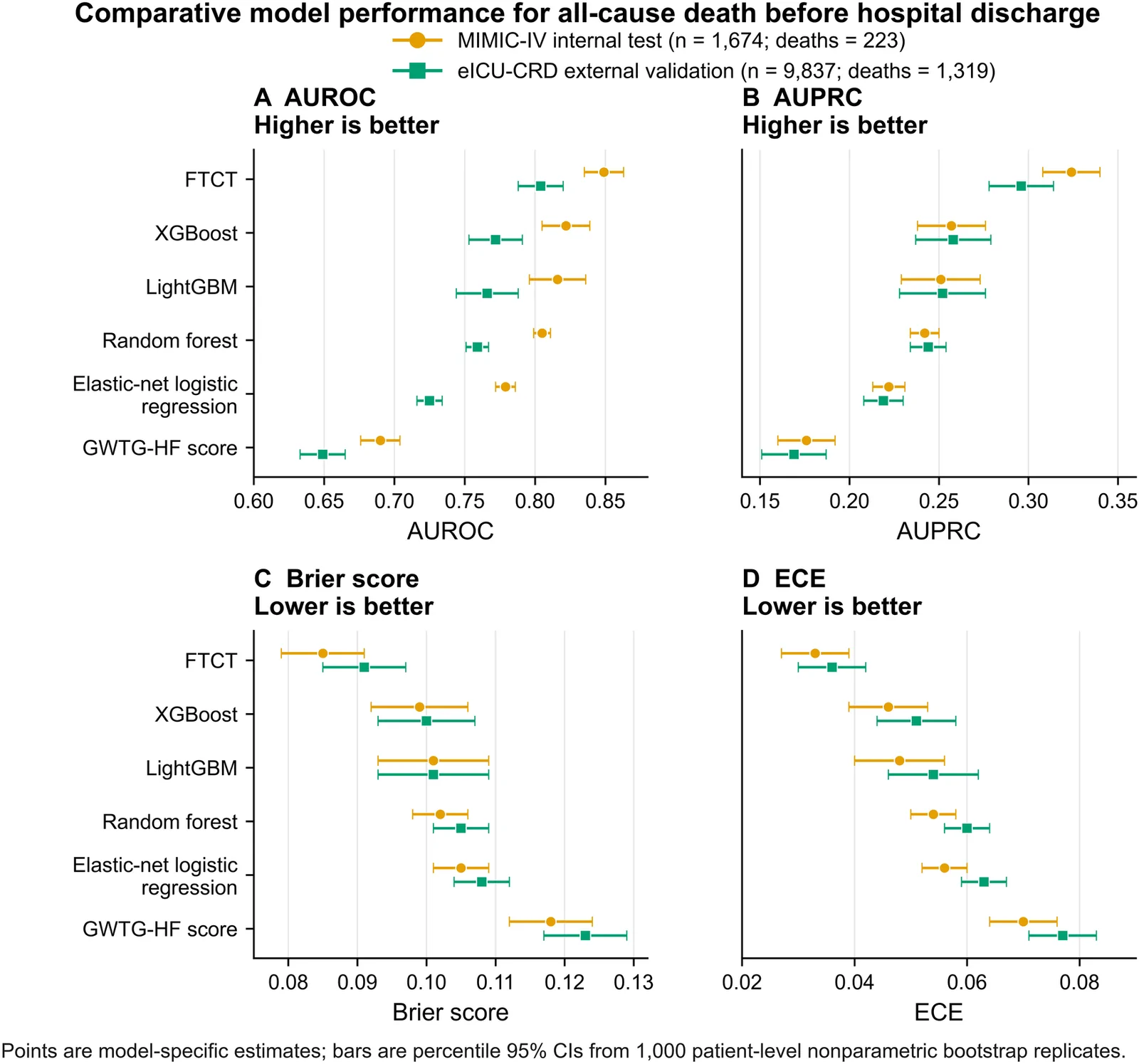
Comparative performance in the MIMIC-IV internal test cohort (*n* = 1,674; 223 in-hospital deaths) and eICU-CRD external validation cohort (*n* = 9,837; 1,319 in-hospital deaths). Points show model-specific AUROC, AUPRC, Brier score, and ECE estimates; horizontal bars show patient-level nonparametric percentile-bootstrap 95% confidence intervals. Lower Brier score and ECE indicate better probability performance. Estimates are model-specific; paired FTCT–XGBoost differences are reported in Supplementary Table S2.

AUROC, AUPRC, Brier score, and ECE 95% CIs were percentile intervals from 1,000 patient-level nonparametric bootstrap replicates; all replicates were successful. ECE used 10 equal-frequency bins. FTCT–XGBoost paired comparisons used identical patient indices; detailed results are provided in Supplementary Table S2. AUPRC, area under the precision–recall curve; AUROC, area under the receiver operating characteristic curve; ECE, expected calibration error; FTCT, Feature-Token Clinical Transformer; GWTG-HF, Get With The Guidelines-Heart Failure.

### 3.4 *Post hoc* operating profiles and error patterns

In a *post hoc* cohort-specific descriptive operating analysis targeting approximately 85% specificity (OP85SPEC), the reported metrics were similar in the two held-out cohorts (Table 3 and Figure 6). In the MIMIC-IV internal test cohort, the model achieved a sensitivity of 0.686 (95% CI: 0.622–0.743), positive predictive value (PPV) of 0.412 (0.363–0.463), negative predictive value (NPV) of 0.946 (0.933–0.957), F1 score of 0.515, and accuracy of 0.828 (0.809–0.845). The corresponding confusion matrix contained 153 true positives, 70 false negatives, 1,233 true negatives, and 218 false positives.

**Table 3:** *Post hoc* cohort-specific operating profiles at approximately 85% specificity.

Dataset	Specificity (95% CI)	Sensitivity (95% CI)	PPV (95% CI)	NPV (95% CI)	F1 score	Accuracy (95% CI)
MIMIC-IV internal test	0.850 (0.830–0.867)	0.686 (0.622–0.743)	0.412 (0.363–0.463)	0.946 (0.933–0.957)	0.515	0.828 (0.809–0.845)
eICU-CRD external validation	0.850 (0.842–0.857)	0.662 (0.636–0.687)	0.406 (0.385–0.427)	0.942 (0.937–0.947)	0.503	0.825 (0.817–0.832)

**Figure 6:**
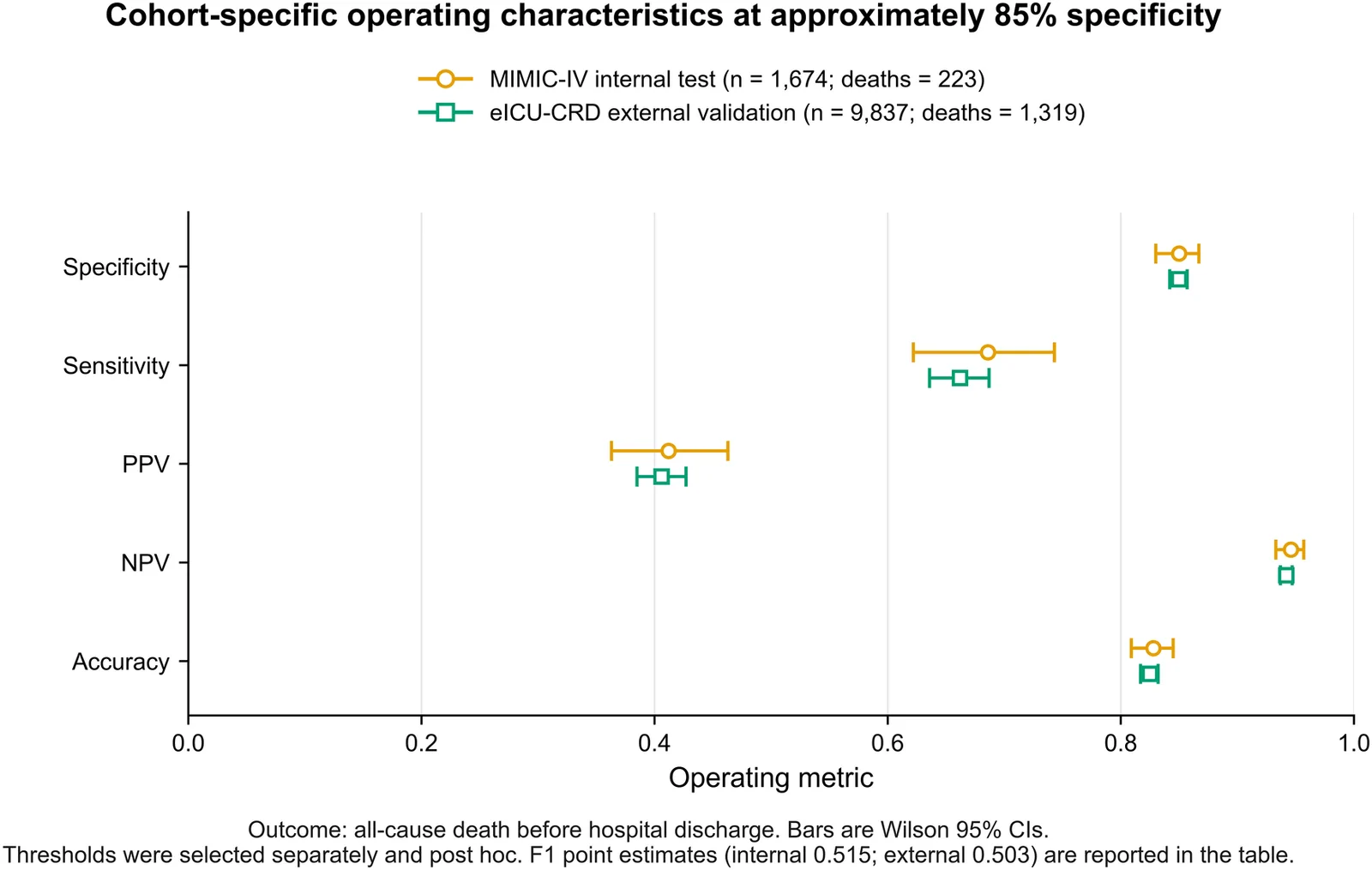
*Post hoc* cohort-specific operating characteristics of FTCT for all-cause death before hospital discharge at approximately 85% specificity. The MIMIC-IV internal test cohort included 1,674 patients and 223 deaths; the eICU-CRD external validation cohort included 9,837 patients and 1,319 deaths. Thresholds were selected separately within each cohort. Error bars are Wilson 95% confidence intervals for sensitivity, specificity, PPV, NPV, and accuracy; F1 scores are point estimates. These results do not represent fixed-threshold external validation performance.

A similar pattern was observed in eICU-CRD, with a sensitivity of 0.662 (0.636–0.687), PPV of 0.406 (0.385–0.427), NPV of 0.942 (0.937–0.947), F1 score of 0.503, and accuracy of 0.825 (0.817–0.832). Of the 1,319 deaths in eICU-CRD, FTCT identified 873 as positive-risk cases and missed 446, while correctly classifying 7,240 of 8,518 survivors and producing 1,278 false positives. At the *post hoc* cohort-specific thresholds, PPV, NPV, and accuracy were similar across the two databases. This profile descriptively combines approximately 85% specificity with lower sensitivity and should not be interpreted as evidence of clinical utility or as a deployment-ready decision threshold.

Figure 6 visualizes the operating metrics and their associated confidence intervals. The small internal-to-external differences in sensitivity (0.686–0.662), PPV (0.412–0.406), NPV (0.946–0.942), F1 score (0.515–0.503), and accuracy (0.828–0.825) indicate similar descriptive performance at the *post hoc*, separately selected cohort-specific thresholds. Because each cohort used its own threshold to target 85% specificity, these results describe the resulting operating profiles and should not be interpreted as validation of a single generalizable deployment threshold.

Thresholds were selected *post hoc* and separately within each cohort to achieve approximately 85% specificity. Wilson CIs are shown for sensitivity, specificity, PPV, NPV, and accuracy; F1 is reported as a point estimate. These results do not represent fixed-threshold external validation performance.

### 3.5 Controlled FT-transformer reference, architecture ablation, robustness, sensitivity, and efficiency

A matched FT-Transformer-style controlled baseline achieved an external validation AUROC of 0.781 (95% CI, 0.760–0.801), AUPRC of 0.282 (0.255–0.309), Brier score of 0.100 (0.091–0.110), and ECE of 0.042 (0.032–0.053) (Table 4 and Figure 7A). The external validation ablation analyses showed descriptive performance changes after modification of the proposed representation (Table 4 and Figure 7A). Removing feature-token encoding caused the largest deterioration, reducing AUROC from 0.804 to 0.773 (95% CI: 0.754–0.792), AUPRC from 0.296 to 0.276, and increasing the Brier score from 0.091 to 0.103. Removing clinical-group embeddings produced the next largest AUROC loss, reducing AUROC to 0.786, a change of −0.018. Reducing the encoder from four to two blocks, removing the nursing-monitoring group, removing the missingness embedding, and replacing the learned risk token with mean pooling produced smaller but directionally consistent losses. This ordering was descriptively consistent with greater sensitivity to removal of feature-token encoding, but it does not establish a causal mechanism or superiority over untested architectures.

**Table 4:** Controlled FT-transformer reference, architecture ablation, robustness, sensitivity, and efficiency analyses.

Block	Setting	Metrics	AUROC change/rationale
Controlled reference	FT-Transformer-style baseline	AUROC: 0.781 (0.760–0.801) AUPRC: 0.282 (0.255–0.309) Brier: 0.100 (0.091–0.110) ECE: 0.042 (0.032–0.053)	Change: −0.023 Matched controlled reference
Ablation	Without feature-token encoding	AUROC: 0.773 (0.754–0.792) AUPRC: 0.276 (0.251–0.301) Brier: 0.103 (0.095–0.111) ECE: 0.044 (0.035–0.053)	Change: −0.031 Prespecified module test
Ablation	Without clinical-group embeddings	AUROC: 0.786 (0.765–0.807) AUPRC: 0.286 (0.259–0.313) Brier: 0.097 (0.087–0.107) ECE: 0.040 (0.029–0.051)	Change: −0.018 Prespecified module test
Ablation	Two Transformer blocks instead of four	AUROC: 0.790 (0.770–0.810) AUPRC: 0.289 (0.263–0.315) Brier: 0.096 (0.087–0.105) ECE: 0.039 (0.029–0.049)	Change: −0.014 Prespecified module test
Ablation	Without missingness embedding	AUROC: 0.795 (0.776–0.814) AUPRC: 0.292 (0.267–0.317) Brier: 0.095 (0.087–0.103) ECE: 0.038 (0.029–0.047)	Change: −0.009 Prespecified module test
Ablation	Without nursing-monitoring group	AUROC: 0.792 (0.771–0.813) AUPRC: 0.288 (0.261–0.315) Brier: 0.097 (0.087–0.107) ECE: 0.040 (0.029–0.051)	Change: −0.012 Prespecified module test
Ablation	Mean pooling instead of risk token	AUROC: 0.798 (0.779–0.817) AUPRC: 0.292 (0.267–0.317) Brier: 0.095 (0.087–0.103) ECE: 0.039 (0.030–0.048)	Change: −0.006 Prespecified module test
Robustness/sensitivity	MIMIC-IV/eICU-CRD common-feature intersection	AUROC: 0.794 (0.775–0.813) AUPRC: 0.288 (0.263–0.313) Brier: 0.094 (0.086–0.102) ECE: 0.040 (0.031–0.049)	Change: −0.010 Frozen-model stress test
Robustness/sensitivity	20% stricter variable-missingness threshold	AUROC: 0.787 (0.767–0.807) AUPRC: 0.284 (0.258–0.310) Brier: 0.095 (0.086–0.104) ECE: 0.041 (0.031–0.051)	Change: −0.017 Frozen-model stress test
Robustness/sensitivity	First 12 h observation window	AUROC: 0.775 (0.755–0.795) AUPRC: 0.276 (0.250–0.302) Brier: 0.099 (0.090–0.108) ECE: 0.045 (0.035–0.055)	Change: −0.029 Frozen-model stress test
Robustness/sensitivity	First 6 h observation window	AUROC: 0.762 (0.744–0.780) AUPRC: 0.269 (0.245–0.293) Brier: 0.104 (0.097–0.111) ECE: 0.046 (0.038–0.054)	Change: −0.042 Frozen-model stress test
Robustness/sensitivity	15% artificial observation masking	AUROC: 0.790 (0.770–0.810) AUPRC: 0.287 (0.261–0.313) Brier: 0.095 (0.086–0.104) ECE: 0.038 (0.028–0.048)	Change: −0.014 Frozen-model stress test
Robustness/sensitivity	Equal-hospital-weighted hospital-cluster bootstrap	AUROC: 0.788 (0.770–0.806) AUPRC: 0.285 (0.261–0.309) Brier: 0.095 (0.088–0.102) ECE: 0.041 (0.033–0.049)	Change: −0.016 Frozen-model stress test
Efficiency	Parameters	0.82 million	Fixed architecture accounting
Efficiency	MACs	18.7 million per patient	Fixed architecture accounting
Efficiency	Model-forward latency, batch size 1	0.42 ms (IQR, 0.39–0.45)	NVIDIA A100 40-GB; model-only benchmark
Efficiency	Model-forward latency, batch size 1,000	9.4 ms (IQR, 9.1–9.8)	Batched inference; approximately 106,000 records/s

**Figure 7:**
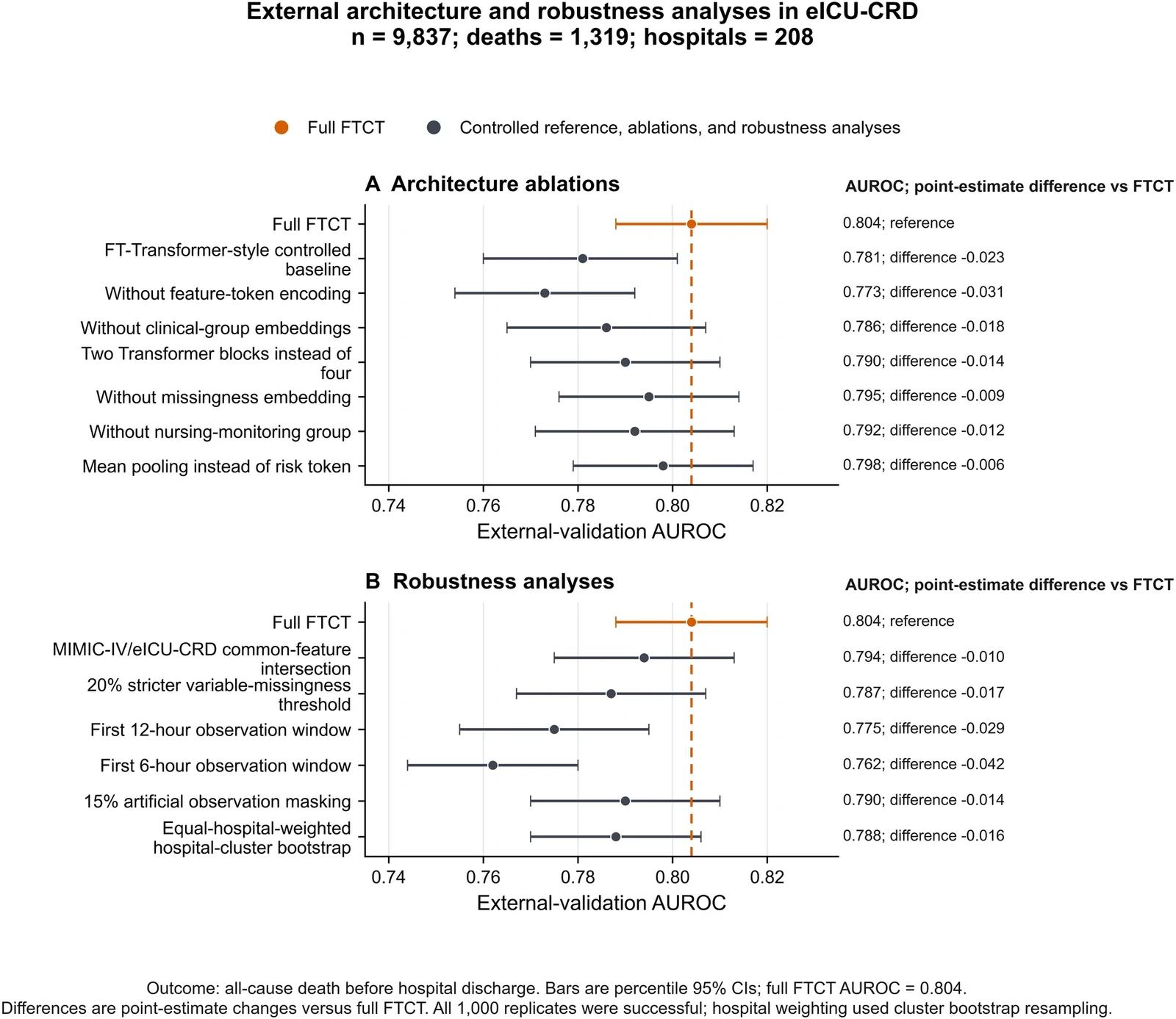
External validation architecture and robustness analyses for all-cause death before hospital discharge in eICU-CRD (*n* = 9,837; 1,319 deaths; 208 hospitals). **(A)** The matched FT-Transformer-style controlled baseline and FTCT architecture ablations. **(B)** Robustness and sensitivity analyses. Points show AUROC estimates; horizontal bars show percentile 95% confidence intervals from 1,000 patient-level bootstrap replicates, except the equal-hospital-weighted analysis, which used 1,000 hospital-cluster replicates. All replicates were successful. The vertical reference line indicates the full FTCT AUROC of 0.804.

Frozen-model stress tests characterized robustness under prespecified input and cohort perturbations (Table 4 and Figure 7B). Restricting inputs to the cross-database common-feature intersection reduced AUROC by 0.010, while masking 15% of observations reduced AUROC by 0.014. A 20% stricter variable-missingness threshold and equal-hospital-weighted hospital-cluster bootstrap resampling produced AUROC changes of −0.017 and −0.016, respectively. When the observation window was shortened, AUROC decreased to 0.775 for the first 12 h and 0.762 for the first 6 h. These changes were accompanied by deterioration in AUPRC, Brier score, and ECE. The larger AUROC losses after window truncation suggest that information collected across the complete first-24-h observation window contributed to model performance and was not fully captured during the first several hours after ICU admission. Under equal-hospital weighting, FTCT achieved an AUROC of 0.788 (95% CI: 0.770–0.806); this modest attenuation suggested some sensitivity to hospital composition without indicating that the pooled result was driven solely by the largest hospitals.

The fixed FTCT implementation contained 0.82 million parameters and required 18.7 million multiply–accumulate operations per patient. Median model-forward latency was 0.42 ms (IQR: 0.39–0.45 ms) for one preprocessed record and 9.4 ms (IQR: 9.1–9.8 ms) for a batch of 1,000 preprocessed records. These measurements excluded data extraction, preprocessing, disk input/output, host-to-device transfer, and CPU time and therefore do not represent end-to-end clinical workflow latency.

All estimates were evaluated in the eICU-CRD external validation cohort (*n* = 9,837; 1,319 in-hospital deaths; 208 hospitals). No model was trained, refitted, or recalibrated using eICU-CRD data. AUROC change is relative to the full FTCT model (0.804). Model-specific 95% CIs are percentile intervals from 1,000 patient-level bootstrap replicates; the equal-hospital-weighted analysis used 1,000 hospital-cluster replicates. All replicates were successful.

## 4 Discussion

In this retrospective secondary analysis of deidentified, credentialed-access data, we developed and externally validated FTCT for 24-h landmark prediction of in-hospital mortality among critically ill adults with heart failure. The model was trained and selected exclusively in MIMIC-IV and then transferred without refitting to the multicenter eICU-CRD cohort. FTCT achieved the most favorable point estimates among the evaluated approaches, including tree-based models, a regularized linear model, and a clinical score, with internal and external AUROCs of 0.849 and 0.804, respectively. Its external AUPRC, Brier score, and ECE estimates were also favorable, and the *post hoc* cohort-specific 85%-specificity profiles produced similar descriptive sensitivity, positive predictive value, negative predictive value, F1 score, and accuracy across the two databases. The largest ablation loss was observed when feature-token encoding was removed, which was descriptively consistent with a contribution from feature-token representation; however, the ablation analysis alone cannot exclude contributions from optimization or model size. Patient-paired bootstrap intervals supported more favorable performance than XGBoost in both held-out cohorts, while comparisons with the remaining models were descriptive.

Feature tokenization is established in FT-Transformer and related tabular architectures and is not claimed as the novelty of FTCT. The principal contribution is the combination of clinical-group embeddings, explicit missingness representation, group-aware pooling, and frozen cross-database evaluation. Although flat tabular models can capture nonlinearities, they receive a single undifferentiated feature vector and must infer structure through model splits, coefficients, or hidden units. FTCT instead encodes creatinine, urine output, vasopressor exposure, mechanical ventilation, and all other retained variables as distinct tokens, each annotated with variable-identity and clinical-group information. Self-attention can then model each field in the patient-specific context supplied by the other fields. The observed ablation pattern was consistent with this interpretation: removing token encoding produced the largest decrease in model performance, while removing clinical-group embeddings, missingness information, or nursing-monitoring variables, reducing attention depth, and replacing the learned risk token with mean pooling produced smaller but directionally consistent decreases. FTCT therefore retains an explicit mapping between the learned tokens and the corresponding variables or clinical groups. This property should be described as architectural traceability rather than as evidence of causal or bedside interpretability. The matched FT-Transformer-style baseline provided a controlled architecture-level comparison and had a lower external AUROC point estimate than FTCT.

These findings extend earlier studies using heart-failure data from deidentified critical-care databases. Gradient-boosted algorithms have performed well for ICU mortality prediction and therefore remain appropriate comparators (9–11). Aykut et al. found that GWTG-HF discriminated short-term mortality better than ADHERE CART but that both scores had limited performance for ICU-related outcomes, supporting its use as a clinical comparator while motivating dynamic, context-aware assessment. Multimodal studies have demonstrated the value of admission notes in addition to structured variables (12). FTCT addresses a narrower but practically relevant setting: hospitals that can reliably extract structured measurements, treatment variables, support interventions, and nurse-charted monitoring variables but cannot assume consistent access to narrative text. The external AUROC difference relative to XGBoost was modest but supported by a patient-paired bootstrap interval, and the same direction of difference was broadly observed across AUROC, AUPRC, Brier score, and ECE. This multidimensional evaluation is important because a model that ranks patients well but produces poorly calibrated probabilities may be difficult to use for threshold-based clinical review (29–31).

From a clinical perspective, FTCT should be interpreted as a retrospective 24-h landmark mortality-risk model rather than as a validated clinical decision-support tool. The external NPV of 0.942, sensitivity of 0.662, and PPV of 0.406 were obtained at a *post hoc* cohort-specific threshold and should not be used to infer workload reduction, rule-out capability, or deployment performance. These estimates should be interpreted in the context of the approximately 13% event prevalence. Prospective workflow and impact studies are required before any use for handoff, escalation review, treatment, or discharge decisions. NRI and decision-curve analysis were not added because validated risk categories, a prespecified deployment threshold range, and a model-triggered intervention were not defined; without this decision context, such analyses could overstate clinical utility.

Several limitations warrant consideration. First, the retrospective design cannot establish whether model-supported review improves clinical outcomes. Second, requiring a complete 24-h observation window defines a landmark cohort and may exclude early deaths, rapid transfers, and short uncomplicated stays; therefore, FTCT should not be interpreted as an admission-time warning system. Third, heart failure was identified retrospectively from diagnosis fields and codes that were not necessarily available at the 24-h landmark, without reliable separation of reduced- vs. preserved-ejection-fraction phenotypes or acute vs. chronic presentations. Fourth, MIMIC-IV (2008–2019) and eICU-CRD (2014–2015) partially overlap in calendar time; thus, the external evaluation was cross-institutional rather than forward temporal validation, and changes in case mix, coding, treatment, and ICU workflows may cause temporal drift. Fifth, the databases shared no source institution or analytic patient record, but deidentification prevented identity-level linkage across different hospitals. Sixth, the frozen model was evaluated without eICU-CRD recalibration, which strengthens assessment of unadapted transportability but leaves calibration shift uncorrected. Both databases represent US critical-care environments, and broader geographic, prospective, and workflow validation remains necessary. Future work should evaluate local recalibration, prospective silent validation, clinician interaction, workload, subgroup-specific performance, and whether risk presentation improves handoff communication without increasing alert burden. These studies are needed because external validation, calibration, and human-centered implementation represent distinct requirements for clinical AI (24, 25, 32–36).

## 5 Conclusion

FTCT combines established feature-token Transformer modeling with clinical-group embeddings, explicit missingness representation, and group-aware pooling for 24-h landmark in-hospital mortality risk stratification among patients with heart failure in intensive and cardiac care units. FTCT showed favorable internal and multicenter external performance and patient-paired differences relative to XGBoost in the evaluated cohorts. Comparisons with the remaining models were descriptive, and the controlled FT-Transformer-style comparison does not establish universal architectural superiority. These findings warrant further prospective evaluation, while clinical utility, workflow benefit, and deployment performance remain to be established through recent-cohort validation, local calibration, and prospective workflow studies.

## Data Availability

Publicly available datasets were analyzed in this study. This data can be found here: MIMIC-IV version 2.2: https://doi.org/10.13026/6mm1-ek67 (Accession No. MIMIC-IV v2.2) eICU-CRD version 2.0: https://doi.org/10.13026/C2WM1R (Accession No. eICU-CRD v2.0).
