# Supplementary material for: Feature-token clinical transformer for 24-h landmark mortality risk stratification in critically ill patients with heart failure: development and multicenter external validation
Source: Front Cardiovasc Med. 2026 Oct 15;13:1945947. doi: 10.3389/fcvm.2026.1945947 (PMC13717714; doi:10.3389/fcvm.2026.1945947)
Supplement: Supplementary file 1 [file Table1.docx]

**Supplementary Material**

# Supplementary Methods

## S1. Bootstrap confidence intervals and paired model comparisons

For AUROC, AUPRC, Brier score, ECE, calibration-in-the-large, and calibration slope, 1,000 patient-level nonparametric bootstrap replicates were drawn separately within each evaluation cohort. Outcomes and predictions remained paired within resampled patients. Percentile 95% confidence intervals were defined by the 2.5th and 97.5th percentiles. A replicate was considered unsuccessful if only one outcome class was present, a metric could not be calculated, or a non-finite estimate was produced; no patient-level replicate failed. FTCT and XGBoost were additionally compared with 1,000 patient-paired replicates using identical patient indices. AUROC and AUPRC differences were FTCT minus XGBoost; Brier score and ECE differences were XGBoost minus FTCT, so positive values favored FTCT. No paired replicate failed.

## S2. Equal-hospital-weighted hospital-cluster bootstrap

The final eICU-CRD cohort comprised 9,837 patients from 208 hospitals. In each of 1,000 replicates, 208 hospitals were sampled with replacement and all eligible patients within each selected hospital were retained together. A hospital selected multiple times contributed proportionally multiple times. Inverse hospital-size patient weights gave every sampled hospital equal total weight, and no additional patient-level resampling was performed within hospitals. This approach preserves within-hospital dependence and tests whether pooled performance is dominated by high-volume hospitals. All replicates were successful. The analysis evaluates sensitivity to hospital composition and size; it is not leave-one-hospital-out or unseen-hospital validation.

## S3. Inference-latency benchmark

The frozen FTCT model was benchmarked on one NVIDIA A100 40-GB GPU using Python 3.10, PyTorch 2.1.2+cu121, CUDA 12.1, and FP16 automatic mixed precision. Model-ready tensors had completed harmonization, imputation, and scaling and were resident in GPU memory before timing. The host CPU prepared batches and launched CUDA operations but was excluded from the synchronized interval. After 50 untimed warm-up iterations, 200 forward passes were measured using synchronized CUDA events for batch sizes 1 and 1,000. Database querying, feature extraction, preprocessing, disk input/output, and host-to-device transfer were excluded. Median latency was 0.42 ms (IQR, 0.39–0.45) for batch size 1 and 9.4 ms (IQR, 9.1–9.8) for batch size 1,000.

**Supplementary Table S1. Database-specific heart-failure cohort definitions and temporal use of diagnosis information**

| **Database / source field** | **Eligible heart-failure definition** | **Diagnosis position** | **Availability relative to 24-hour landmark** |
| --- | --- | --- | --- |
| MIMIC-IV; mimiciv_hosp.diagnoses_icd | ICD-9-CM 428.0, 428.1, 428.20–428.23, 428.30–428.33, 428.40–428.43, and 428.9; or ICD-10-CM I50.1, I50.20–I50.23, I50.30–I50.33, I50.40–I50.43, I50.810–I50.814, I50.82–I50.84, I50.89, and I50.9 | Any sequence number; not restricted to the principal diagnosis | Hospital-level administrative diagnoses may be assigned after the 24-hour landmark and were used only for retrospective cohort eligibility, not as predictors |
| eICU-CRD; diagnosis table | ICD-9-CM category 428 (all subcodes listed above) or explicit active-diagnosis labels indicating heart failure, congestive heart failure, or CHF | Primary, Major, or Other | Diagnosis entries could be recorded during the ICU stay and were used only for retrospective cohort eligibility, not as predictors |

**Supplementary Table S2. Patient-paired performance differences between FTCT and XGBoost**

| **Cohort** | **Metric** | **Difference direction** | **Difference (percentile 95% CI)** |
| --- | --- | --- | --- |
| MIMIC-IV internal test | AUROC | FTCT − XGBoost | 0.027 (0.011–0.043) |
| MIMIC-IV internal test | AUPRC | FTCT − XGBoost | 0.067 (0.049–0.085) |
| MIMIC-IV internal test | Brier score | XGBoost − FTCT | 0.014 (0.007–0.021) |
| MIMIC-IV internal test | ECE | XGBoost − FTCT | 0.013 (0.006–0.020) |
| eICU-CRD external validation | AUROC | FTCT − XGBoost | 0.032 (0.014–0.050) |
| eICU-CRD external validation | AUPRC | FTCT − XGBoost | 0.038 (0.018–0.058) |
| eICU-CRD external validation | Brier score | XGBoost − FTCT | 0.009 (0.002–0.016) |
| eICU-CRD external validation | ECE | XGBoost − FTCT | 0.015 (0.008–0.022) |

**Supplementary Table S3. Calibration and predicted-risk distribution summaries**

| **Cohort** | **n / deaths** | **Calibration-in-the-large (95% CI)** | **Calibration slope (95% CI)** | **Mean predicted / observed risk** | **Median (IQR); 5th–95th percentile** |
| --- | --- | --- | --- | --- | --- |
| MIMIC-IV internal test | 1,674 / 223 | −0.164 (−0.355 to 0.023) | 0.767 (0.673–0.885) | 0.146 / 0.133 | 0.047 (0.010–0.181); 0.001–0.715 |
| eICU-CRD external validation | 9,837 / 1,319 | 0.297 (0.215–0.376) | 0.609 (0.567–0.649) | 0.113 / 0.134 | 0.031 (0.007–0.120); 0.001–0.622 |

**Supplementary Table S4. Cross-database implementation of GWTG-HF variables**

| **GWTG-HF component** | **Harmonized definition** | **Unit or missing-data rule** |
| --- | --- | --- |
| Age | Age at ICU admission | Years; no cross-database recoding beyond eligibility checks |
| Systolic blood pressure | Earliest valid value during the first 24 hours after ICU admission | mmHg |
| Heart rate | Earliest valid value during the first 24 hours after ICU admission | beats/min |
| Blood urea nitrogen | Earliest valid value during the first 24 hours after ICU admission | mg/dL |
| Serum sodium | Earliest valid value during the first 24 hours after ICU admission | mmol/L |
| COPD history | Database-specific diagnosis or history fields harmonized to present/absent | Missing values handled by the training-set-fitted procedure |
| Race/ethnicity | Database categories harmonized as Black versus non-Black | Unknown or indeterminate entries treated as missing, not automatically assigned |
